# Large-deformation consolidation analysis of foundations based on the elliptical cylinder equivalent drainage plate theory

**DOI:** 10.1371/journal.pone.0352168

**Published:** 2026-06-25

**Authors:** Aiwu Yang, Tianli Liu, Shaopeng Yang

**Affiliations:** Department of Civil Engineering, College of Environmental Science and Engineering, Donghua University, Shanghai, China; Henan Polytechnic University, CHINA

## Abstract

Building upon the elliptical cylinder equivalence model, this study integrates the nonlinear variations in soil compressibility and permeability, together with the attenuation behavior of vacuum pressure, to develop large-deformation consolidation models that account for both vacuum loss and the effective influence zone at the base of drainage plates. Corresponding numerical solutions are derived. The results demonstrate that the model incorporating the bottom influence zone exhibits close agreement with experimental data from capped drainage plate tests, validating the rationality and applicability of the proposed consolidation framework and its numerical implementation. In contrast, the model considering only vacuum loss yields greater deviations relative to the experimental results. This finding indicates that neglecting the bottom influence zone of the drainage plate leads to an underestimation of total settlement, thereby misrepresenting the long-term consolidation behavior of ultra-soft soil foundations.

## Introduction

The theory of foundation consolidation is a fundamental component in geotechnical engineering design, construction, and long-term performance evaluation. Since its initial formulation by Terzaghi [1] in 1924, nearly a century of research and practical application has refined and expanded the theory’s conceptual and analytical framework. Drawing upon numerous engineering case studies, researchers have continually improved the understanding of soil behavior under consolidation, thereby establishing a solid foundation for the continued development of consolidation theory.

At present, consolidation analysis of soft soil foundations reinforced with prefabricated vertical drains (PVDs) predominantly relies on the classical sand drain consolidation theory [2–4]. However, this approach simplifies PVDs into idealized cylindrical drainage elements, which significantly deviates from their actual rectangular geometry. Given that PVDs typically possess a width-to-thickness ratio ranging from 25 to 50 [5,6], this simplification introduces substantial discrepancies, leading to inaccurate predictions of field consolidation behavior. To mitigate these errors, various equivalence methods—such as perimeter equivalence, area equivalence, and equivalent diameter formulations—have been proposed. Nevertheless, their performance varies across soil types and loading conditions, and no universal consensus has been achieved [7].

To address this limitation, Huang, et al. [8,9] and Tian, et al. [10,11] introduced an elliptical cylinder equivalence method, representing PVDs as flattened elliptical drainage bodies. This approach more accurately captures the geometric “shape effect” of the drainage element. Subsequent refinements incorporated considerations of well resistance, vacuum pressure transmission, and the nonlinear dependency of soil permeability and compressibility on stress and void ratio, thereby progressively improving the fidelity of the consolidation model.

Ultra-soft soils, owing to their distinctive composition, exhibit the so-called “three-high” characteristics—high water content, high compressibility, and high void ratio—making them particularly susceptible to large deformations under external loading [12]. Experimental investigations by ZengLingling, et al. [13] revealed a pronounced linear relationship between the permeability coefficient and void ratio in a double-logarithmic scale for highly compressible soft soils. Consequently, employing conventional nonlinear permeability models to describe vacuum preloading consolidation in ultra-soft foundations may lead to significant underestimation of final settlements, compromising the accuracy of engineering design and performance predictions.

In addition, the distribution of vacuum pressure in PVD systems typically exhibits a linear decay with depth along the drain, while demonstrating a nonlinear attenuation with increasing radial distance in the surrounding soil. CaiYuanqiang, et al. [14] developed a mathematical model to describe this behavior—linear decay within the drain and nonlinear decay in the adjacent soil—but the model remains parameter-intensive and computationally complex, as it is grounded in the classical sand drain framework. Similarly, Wang, et al. [15], Chai, et al. [16], Indraratna, et al. [17,18], and Wang, et al. [19] incorporated vacuum pressure attenuation patterns into large-strain consolidation models for soft soils, providing valuable insights into vacuum preloading behavior.

Furthermore, empirical evidence indicates that when PVDs are installed to shallow depths, an effective influence zone develops beneath the drain tip due to localized consolidation and stress redistribution. However, most existing models treat such scenarios as partially penetrated or layered systems, without explicitly accounting for the geometry and influence extent of this bottom zone. As a result, notable discrepancies arise between predicted and observed settlements near the drain base.

Despite significant progress in individual aspects of consolidation theory, existing models often address elements such as equivalent geometry, soil nonlinearity, and vacuum pressure attenuation in isolation, or they rely heavily on conventional small-strain assumptions. Furthermore, the localized boundary effects at the drain tip are frequently oversimplified. In light of these limitations, it is necessary to provide a more comprehensive, coupled, and physically rigorous method for predicting the settlement behavior of ultrasoft soil foundations that explicitly incorporates (i) the geometric characteristics of the drainage plate, (ii) the effective influence zone at its bottom, (iii) soil nonlinearity, and (iv) realistic vacuum pressure transmission behavior. The proposed model aims to advance the theoretical framework of vertical drain consolidation and provide a more robust analytical basis for the design and optimization of vacuum preloading treatments in ultra-soft soil foundations.

## List of symbols

For clarity and ease of reference, the main mathematical symbols, parameters, and their corresponding physical definitions used in this large-deformation consolidation model are summarized in Table 1 in the order of their first appearance.

**Table 1:** List of symbles.

Symbol	Description	Unit
a	The focal coordinate of both the family of ellipses and hyperbolas, and also the focal coordinate of the equivalent elliptical cylindrical drainage body.	As detailed in the subsequent text.
η, ξ, z	The fundamental variable in the elliptical cylindrical coordinate system.	
b	The width of the PVD.	
δ	The thickness of the PVD.	
e	The soil void ratio.	
e_0_	The initial void ratio.	
σ′	The effective stress.	
σ′_0_	The initial effective stress.	
I_c_	The soil compression index.	
k_h_	The permeability coefficient.	
k_h0_	The initial permeability coefficient.	
α	The parameter of the permeability model.	
p	The vacuum pressure in the soil.	
p_0_	The vacuum pressure applied at the top of the drainage plate.	
t	The vacuum preloading consolidation time.	
z	The soil depth	
k_1_	The attenuation coefficient of vacuum pressure within the drainage plate.	
k_2_	The attenuation coefficient of vacuum pressure in the soil.	
r	The radial distance from the center of the equivalent drainage plate.	
r_w_	The equivalent radius of the drainage body.	
m, κ	The vacuum pressure growth coefficient.	
L	Equivalent drainage depth.	
l	Vertical influence depth of the PVD.	
r_s_	Radius of the smear zone.	
r_e_	Radius of the influence zone for a single drain.	
k_w_	Permeability coefficient of the equivalent drain.	
k_s_	Horizontal permeability coefficient of the smear zone.	
k_v_	Vertical permeability coefficient of the undisturbed soil.	
u	Excess pore water pressure in the foundation soil.	
ε_v_	The volumetric strain of the soil.	
σ	The total stress of the soil.	
G_s_	The unit weight of the soil particles.	
γ_w_	The unit weight of water.	
ū_r_	The average excess pore water pressure at depth.	
u_r_	The local excess pore water pressure at the same depth.	
a cosh	The inverse hyperbolic cosine function.	
u_w_	The excess pore water pressure within the drain.	
Q_w_	The flow rate within the drainage plate per unit time, and also the magnitude of well resistance inside the plate.	
u_1_	The excess pore water pressure in the upper soil layer.	
ū_1_	The average excess pore water pressure in the upper soil layer.	
Ū_1_	The average degree of consolidation for the upper soil layer.	
ū_2_	The average excess pore water pressure in the lower soil layer.	
C_v_	The vertical coefficient of consolidation.	
F	The effective influence zone at the bottom of the drainage plate.	
T_v_	The vertical consolidation time factor.	
m	The boundary parameter.	
Ū_2_	The average degree of consolidation for the lower soil layer.	
Ū	The total average degree of consolidation of the entire foundation.	

## Establishment of the consolidation calculation model

### Elliptical cylindrical coordinate system

**Fig 1:**
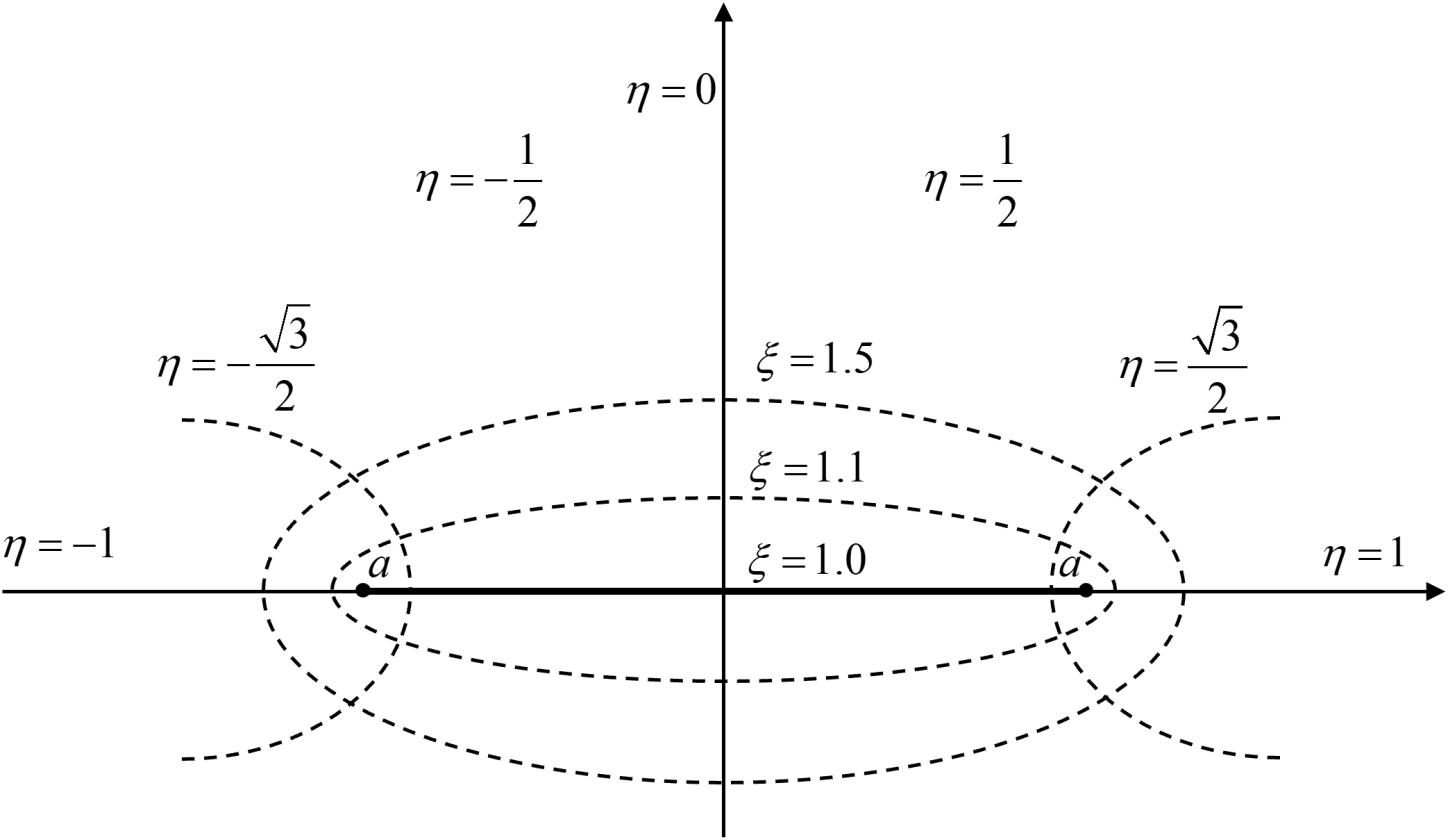
Sketch of elliptic coordinate system.


Based on the elliptical cylindrical coordinate system theory proposed by Wang Zhuxi et al. [20] (see Eq 1), the following coordinate transformation is established, as illustrated in Fig 1.

$$\begin{array}{c} \left\{ \begin{array}{c} x=a\eta \xi \\ y=\pm a\sqrt{\left(\xi^{\text{2}}-\text{1}\right)\left(\text{1}-\eta^{\text{2}}\right)}\\ z=z \end{array}\right.. \end{array}$$ (1)

In this equation: *a* denotes the focal coordinate of both the family of ellipses and hyperbolas, and also represents the focal coordinate of the equivalent elliptical cylindrical drainage body; *η*, *ξ*, *z* are the fundamental variable in the elliptical cylindrical coordinate system.

### Elliptical cylinder equivalence method

**Fig 2:**
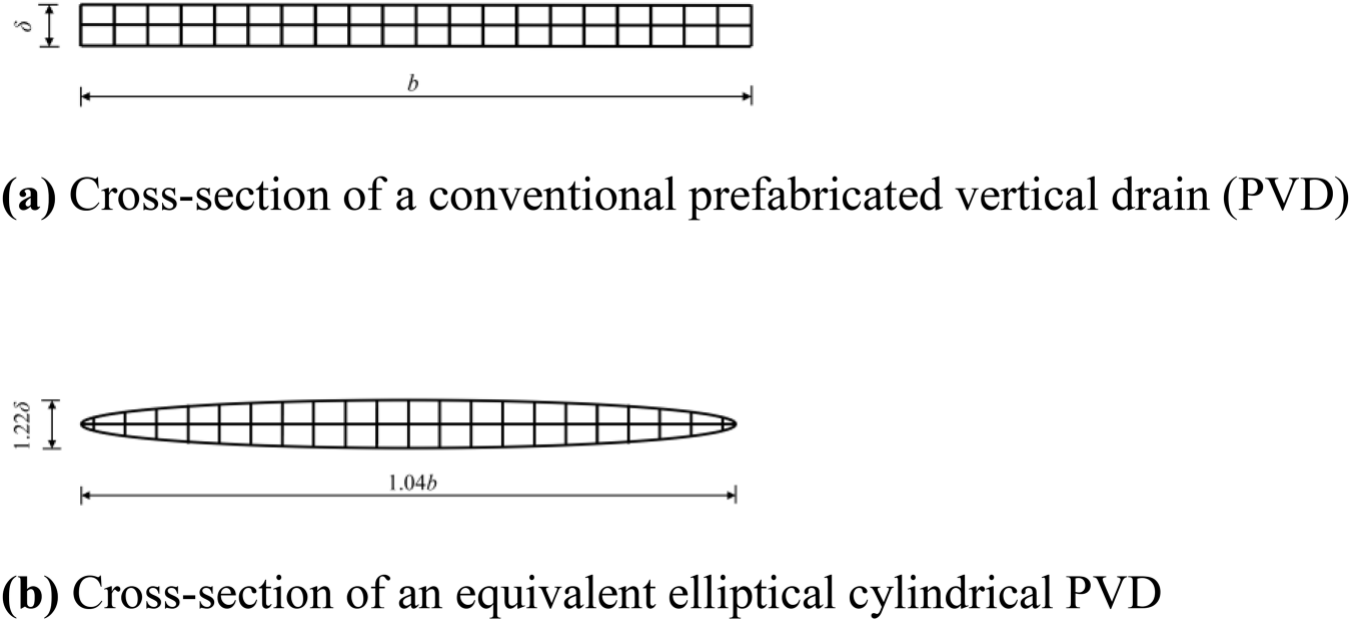
Equivalent model of drainage board.

Huang Chaoxuan et al. [7] proposed an approach that treats the prefabricated vertical drain (PVD) as a flattened elliptical cylinder, which more accurately represents its actual geometry in engineering applications. Based on the principle of optimal equivalence in both area and perimeter, the equivalent elliptical cylinder can be defined with a major axis of 1.04*b* and a minor axis of 1.22*δ*, as shown in Fig 2. Here, *b* denotes the width of the PVD, and *δ* its thickness, with *b* being much greater than *δ*.

Specifically, these two coefficients (1.04 and 1.22) are derived by simultaneously satisfying the equivalence of the cross-sectional area and the perimeter between the physical rectangular PVD and the theoretical elliptical cylinder. This dual-equivalence approach ensures that the model preserves both the cross-sectional flow capacity (well resistance) and the lateral surface area for seepage (contact area with surrounding soil). By integrating these geometric parameters, the elliptical cylinder model provides a more consistent and rigorous representation of the PVD’s hydraulic performance compared to traditional circular equivalence methods.

### Soil compressibility and permeability characteristics

**Fig 3:**
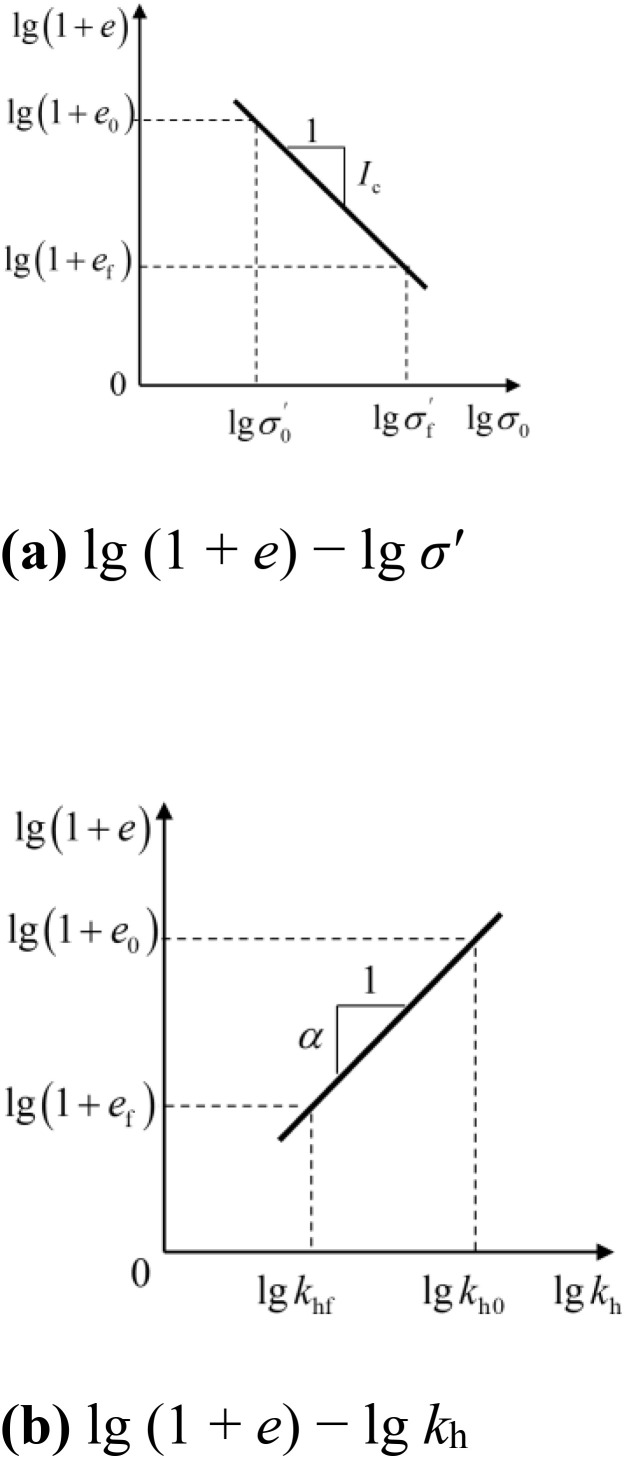
Schematic curve of soil compression versus infiltration properties.


Butterfield [21] and XueQing, et al. [22] experimentally demonstrated that highly compressible silts exhibit a pronounced linear correlation between void ratio and effective stress when plotted in a double-logarithmic coordinate system, as expressed in Eq (2). Building upon this, ZengLingling, et al. [13] further observed that for soils undergoing strains greater than 20%, the permeability coefficient and void ratio also display a strong linear relationship on a double-logarithmic scale, as shown in Eq (3). The interrelationships between soil compressibility and permeability characteristics are illustrated schematically in Fig 3.

$$\begin{array}{c} \frac{\text{1}+e}{\text{1}+e_{\text{0}}}={\left(\frac{\overset{\prime }{\underset{\text{0}}{\sigma }}}{\sigma^{\prime }}\right)}^{I_{\text{c}}} \end{array}$$ (2)

$$\begin{array}{c} \frac{k_{\text{h}}}{k_{\text{h}\text{0}}}={\left(\frac{\text{1}+e}{\text{1}+e_{\text{0}}}\right)}^{\alpha } \end{array}$$ (3)

In these equations: *e* denotes the soil void ratio; *e*_0_ is the initial void ratio; *σ′* is the effective stress; *σ′*_0_ is the initial effective stress; *I*_c_ is the soil compression index; *k*_h_ is the permeability coefficient; *k*_h0_ is the initial permeability coefficient; and *α* is the parameter of the permeability model.

Compressibility (represented by the soil compression index *I*_c_) and permeability (represented by the permeability model parameter *α*) are two major coupled factors governing the engineering properties of soils, both of which heavily depend on soil composition and structure (represented by the soil void ratio *e*) [23,24]. In the proposed consolidation framework, the soil compression index *I*_c_ and the permeability parameter *α* are treated as dynamic variables that are linearly related to the void ratio *e*.

### Vacuum pressure attenuation pattern

Indraratna, et al. [25] also reported through experimental investigations that vacuum pressure generally exhibits a nearly linear attenuation trend along the depth of the prefabricated vertical drain (PVD) and proposed an attenuation model, as shown in Eq (4). However, further analysis of test data revealed that, although vacuum pressure decays linearly with depth along the PVD, it varies nonlinearly in the radial direction within the surrounding soil. Thus, Eq (4) cannot accurately represent the experimental observations.

**Fig 4:**
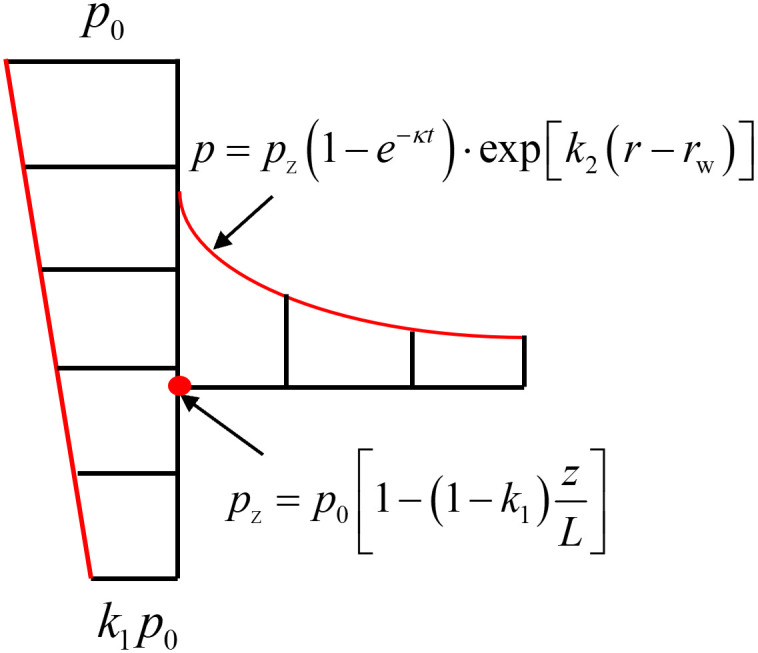
Schematic diagram of vacuum change.


To overcome this limitation, the present study develops a mathematical model (Eq 5) suitable for describing the variation of vacuum pressure in vacuum-preloaded reclaimed soft soil foundations. The variation pattern is schematically illustrated in Fig 4.

$$\begin{array}{c} p=p_{\text{0}}\left[\text{1}-\left(\text{1}-k_{\text{1}}\right)\frac{z}{L}\right] \end{array}$$ (4)

$$\begin{array}{c} p=p_{\text{0}}\left(\text{1}-e^{-\kappa t}\right)\left[\text{1}-\left(\text{1}-k_{\text{1}}\right)\frac{z}{L}\right]\cdot \text{exp}\left[k_{\text{2}}\left(r-r_{\text{w}}\right)\right] \end{array}$$ (5)

In the equation: *p* is the vacuum pressure in the soil (kPa); *p*_0_ is the vacuum pressure applied at the top of the drainage plate (kPa); *t* is the vacuum preloading consolidation time (h); *z* is the soil depth (m); *k*_1_ is the attenuation coefficient of vacuum pressure within the drainage plate; *k*_2_ is the attenuation coefficient of vacuum pressure in the soil; *r* is the radial distance from the center of the equivalent drainage plate (m); *r*_w_ is the equivalent radius of the drainage body (m); and *m*, *κ* are the vacuum pressure growth coefficient.

Physically, the attenuation coefficient *k*_2_ represents the resistance of the soil to the radial propagation of the vacuum pressure. This spatial attenuation behavior is highly dependent on the initial permeability and void ratio of the ultra-soft soil; specifically, a lower initial permeability generally yields a higher resistance to vacuum transfer, thereby intensifying the radial attenuation. Furthermore, for ultra-soft soils with high water content, *k*_2_ is not strictly a static value but typically manifests as a nonlinear dynamic parameter that evolves as the soil skeleton compresses during the consolidation process, which can be calibrated according to specific site conditions and initial permeability characteristics.

### Schematic of the calculation model

**Fig 5:**
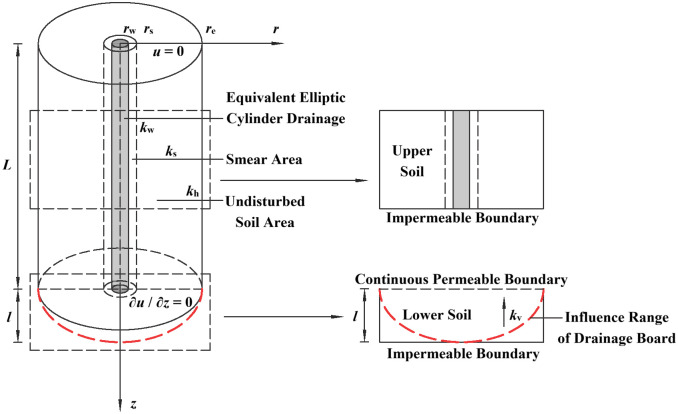
Schematic diagram of the single-drain model considering the bottom influence zone.

The single-drain foundation treatment model considering the effective influence zone at the drain bottom is illustrated in Fig 5. In this model:

*L* is equivalent drainage depth; *l* is vertical influence depth of the PVD; *r*_w_ is equivalent radius of the drain; *r*_s_ is radius of the smear zone; *r*_e_ is radius of the influence zone for a single drain; *k*_w_ is permeability coefficient of the equivalent drain; *k*_s_ is horizontal permeability coefficient of the smear zone; *k*_h_ is horizontal permeability coefficient of the undisturbed soil; *k*_v_ is vertical permeability coefficient of the undisturbed soil; *u* is excess pore water pressure in the foundation soil.

To establish the large-deformation consolidation equation for foundations considering the effective influence zone at the bottom of the PVD, the following basic assumptions are made:

(1) The soil is fully saturated.
(2) Soil particles and pore water are incompressible.
(3) The soil compression coefficient remains constant, and lateral deformation is neglected.
(4) Water flow in the soil satisfies Darcy’s law.
(5) The soil satisfies the equal-strain condition.

It should be emphasized that the current model assumes a continuous permeable boundary at the vertical influence depth *l*, which is consistent with the most common geological conditions in ultra-soft soil reclamation where PVDs are installed into permeable or semi-permeable underlying layers. In this scenario, the ‘bottom influence zone’ effectively contributes to the overall consolidation by allowing for localized stress redistribution and drainage beneath the drain tip. However, if the PVDs are installed in a relatively impermeable clay layer, the vertical drainage path at the base is restricted, and the ‘bottom influence zone’ effect will significantly diminish (can reach over 50% [26]). Therefore, the proposed analytical framework is primarily applicable to foundations where the underlying soil properties support the formation of an effective drainage influence zone at the drainage plate base.

## Derivation of the governing equation

According to Gibson’s one-dimensional large-deformation consolidation theory [27], the relationship between the Lagrangian coordinate and the flow coordinate can be expressed as follows:


$$\begin{array}{c} \frac{\partial \xi }{\partial z}=\frac{\text{1}+e}{\text{1}+e_{\text{0}}} \end{array}$$ (6)

Where *e* = *e* (*z*, *t*), *e* is the soil void ratio; and *e*_0_ = *e*_0_ (*z*, 0), *e*_0_ is the initial void ratio. The relationship between soil strain and void ratio is expressed as:


$$\begin{array}{c} \frac{\partial \varepsilon_{\text{v}}}{\partial t}=-\frac{\text{1}}{\text{1}+e}\frac{\partial e}{\partial t} \end{array}$$ (7)

Where *ε*_v_ is the volumetric strain of the soil.

The stress equilibrium equation of the soil can be written as:


$$\begin{array}{c} \frac{\partial \sigma }{\partial \xi }=-\frac{\left(G_{\text{s}}+e\right)\gamma_{\text{w}}}{\text{1}+e} \end{array}$$ (8)

Where *σ* is the total stress of the soil; *G*_s_ is the unit weight of the soil particles; and *γ*_w_ is the unit weight of water.

Based on the sand drain consolidation theory and the transformation in the elliptical cylindrical coordinate system [28,29], through integral transformation, the fundamental differential equations governing the consolidation of the foundation with an equivalent elliptical cylindrical drain can be derived as follows.

The continuity equations for the smear zone and the undisturbed soil zone are:


$$\begin{array}{c} \frac{\partial u_{\text{r}}}{\partial r}=\frac{a^{\text{2}}\gamma_{\text{w}}}{\text{2}k_{\text{s}}}\left(\frac{r_{\text{e}}\sqrt{\overset{\text{2}}{\underset{\text{e}}{r}}-\text{1}}}{\sqrt{r^{\text{2}}-\text{1}}}-r\right){\left[\frac{\text{1}+e_{\text{0}}}{\text{1}+e}\frac{\partial }{\partial a}\left(\frac{\text{1}+e_{\text{0}}}{\text{1}+e}\frac{k_{\text{v}}}{\gamma_{\text{w}}}\frac{\partial \overset{-}{u_{\text{r}}}}{\partial a}\right)+\frac{\partial \varepsilon_{\text{v}}}{\partial t}\right]}_{\left(r_{\text{w}}\leq r\leq r_{\text{s}}\right)} \end{array}$$ (9)

$$\begin{array}{c} \frac{\partial u_{\text{r}}}{\partial r}=\frac{a^{\text{2}}\gamma_{\text{w}}}{\text{2}k_{\text{h}}}\left(\frac{r_{\text{e}}\sqrt{\overset{\text{2}}{\underset{\text{e}}{r}}-\text{1}}}{\sqrt{r^{\text{2}}-\text{1}}}-r\right){\left[\frac{\text{1}+e_{\text{0}}}{\text{1}+e}\frac{\partial }{\partial a}\left(\frac{\text{1}+e_{\text{0}}}{\text{1}+e}\frac{k_{\text{v}}}{\gamma_{\text{w}}}\frac{\partial \overset{-}{u_{\text{r}}}}{\partial a}\right)+\frac{\partial \varepsilon_{\text{v}}}{\partial t}\right]}_{\left(r_{\text{s}}\leq r\leq r_{\text{e}}\right)} \end{array}$$ (10)

The average excess pore water pressure at any depth in the foundation soil is given by:


$$\begin{array}{c} \overset{-}{u_{\text{r}}}=\frac{\int_{D}u_{\text{r}}\text{ d}A+\int_{E}u_{\text{r}}\text{ d}A}{\pi a^{\text{2}}\left(r_{\text{e}}\sqrt{\overset{\text{2}}{\underset{\text{e}}{r}}-\text{1}}-r_{\text{w}}\sqrt{\overset{\text{2}}{\underset{\text{w}}{r}}-\text{1}}\right)} \end{array}$$ (11)

Where *ū*_r_ is the average excess pore water pressure at depth; and *u*_r_ is the local excess pore water pressure at the same depth.

Substituting Eqs (9) and (10) into Eq (11) yields:


$$\begin{array}{c} \overset{-}{u_{\text{r}}}=F\frac{a^{\text{2}}\gamma_{\text{w}}}{k_{\text{h}}}\left[\frac{\text{1}+e_{\text{0}}}{\text{1}+e}\frac{\partial }{\partial z}\left(\frac{\text{1}+e_{\text{0}}}{\text{1}+e}\frac{k_{\text{v}}}{\gamma_{\text{w}}}\frac{\partial \overset{-}{u_{\text{r}}}}{\partial a}\right)+\frac{\partial \varepsilon_{\text{v}}}{\partial t}\right]+u_{\text{w}} \end{array}$$ (12)

$$\begin{array}{c} \text{Where }F=\frac{\begin{array}{c} \frac{k_{\text{h}}}{k_{\text{s}}}\left[\begin{array}{c} \text{4}\left(\lambda_{\text{e}}-\lambda_{\text{w}}\right)\text{cosh}\left(\text{4}\lambda_{\text{e}}\right)+\text{sinh}\left(\text{4}\lambda_{\text{s}}\right)-\text{sinh}\left(\text{4}\lambda_{\text{w}}\right)\\ -\text{8}\text{cosh}\left(\text{2}\lambda_{\text{s}}\right)\text{sinh}\left(\text{2}\lambda_{\text{e}}\right)+\text{8}\text{cosh}\left(\text{2}\lambda_{\text{w}}\right)\text{sinh}\left(\text{2}\lambda_{\text{e}}\right) \end{array}\right]\\ +\text{4}\left(\lambda_{\text{e}}-\lambda_{\text{s}}\right)\text{cosh}\left(\text{4}\lambda_{\text{e}}\right)-\text{3}\text{sinh}\left(\text{4}\lambda_{\text{e}}\right)-\text{sinh}\left(\text{4}\lambda_{\text{s}}\right)\\ +\text{8}\text{cosh}\left(\text{2}\lambda_{\text{s}}\right)\text{sinh}\left(\text{2}\lambda_{\text{e}}\right) \end{array}}{\text{3}\text{2}\left[\text{sinh}\left(\text{2}\lambda_{\text{e}}\right)-\text{sinh}\left(\text{2}\lambda_{\text{w}}\right)\right]}; \end{array}$$

*λ*_e_ = a cosh *r*_e_; *λ*_w_ = a cosh *r*_w_; *λ*_s_ = a cosh *r*_s_; “a cosh” represents the inverse hyperbolic cosine function; and *u*_w_ denotes the excess pore water pressure within the drain.

It should be noted that while the smear zone radius *r*_s_ and the equivalent drain radius *r*_w_ are defined in the Lagrangian coordinate system, the ratio *r*_s_ / *r*_w_ is adopted based on established empirical standards for mandrel-driven PVDs. Although large strains in ultra-soft soils may induce localized variations in permeability gradients near the interface, the smear effect primarily influences the initial consolidation rate. Within the significant deformation range observed in this study, the assumption of a stable *r*_s_/*r*_w_ ratio remains robust and provides a reliable representation of the horizontal drainage impedance during the long-term consolidation process.

By combining the flow continuity equation at the interface between the drain and the surrounding soil, one obtains:


$$\begin{array}{c} -\frac{\text{1}}{\gamma_{\text{w}}}\frac{\partial }{\partial \xi }\left(Q_{\text{w}}\frac{\partial u_{\text{w}}}{\partial \xi }\right)={\left(\text{2}\pi r_{\text{w}}\frac{k_{\text{s}}}{\gamma_{\text{w}}}\frac{\partial u_{\text{r}}}{\partial r}\right)}_{r=r_{\text{w}}} \end{array}$$ (13)

In the equation: *Q*_w_ represents the flow rate within the drainage plate per unit time, which also reflects the magnitude of well resistance inside the plate.

Substituting Eq (9) into Eq (13) yields:


$$\begin{array}{c} -\frac{\text{1}}{\pi a^{\text{2}}r_{\text{w}}A_{\text{e}}\gamma_{\text{w}}}\frac{\partial }{\partial \xi }\left(Q_{\text{w}}\frac{\partial u_{\text{w}}}{\partial \xi }\right)=\frac{\text{1}+e_{\text{0}}}{\text{1}+e}\frac{\partial }{\partial z}\left(\frac{\text{1}+e_{\text{0}}}{\text{1}+e}\frac{k_{\text{v}}}{\gamma_{\text{w}}}\frac{\partial \overset{-}{u_{\text{r}}}}{\partial z}\right)+\frac{\partial \varepsilon_{\text{v}}}{\partial t} \end{array}$$ (14)

Where $A_{\text{e}}=\frac{r_{\text{e}}\sqrt{\overset{\text{2}}{\underset{\text{e}}{r}}-\text{1}}}{\sqrt{r^{\text{2}}-\text{1}}}-r$.

Combining Eqs (12) and (14) yields:


$$\begin{array}{c} \overset{-}{u_{\text{r}}}=-\frac{F}{\pi r_{\text{w}}A_{\text{e}}k_{\text{h}}}\frac{\partial }{\partial \xi }\left(Q_{\text{w}}\frac{\partial u_{\text{w}}}{\partial \xi }\right)+u_{\text{w}} \end{array}$$ (15)

Combining Eqs (6) and (15), we obtain:


$$\begin{array}{c} \frac{\partial }{\partial z}\left(Q_{\text{w}}\frac{\text{1}+e_{\text{0}}}{\text{1}+e}\frac{\partial u_{\text{w}}}{\partial z}\right)+\frac{\pi r_{\text{w}}A_{\text{e}}k_{\text{h}}}{F}\frac{\text{1}+e}{\text{1}+e_{\text{0}}}\left(\overset{-}{u_{\text{r}}}-u_{\text{w}}\right)=\text{0} \end{array}$$ (16)

The principle of effective stress in the soil is expressed as:


$$\begin{array}{c} \sigma^{\prime }=\overset{\prime }{\underset{\text{0}}{\sigma }}-\overset{-}{u_{\text{r}}} \end{array}$$ (17)

From Eqs (7) and (8), it follows that:


$$\begin{array}{c} \frac{k_{\text{h}}}{k_{\text{h}\text{0}}}={\left(\frac{\sigma^{\prime }}{\overset{\prime }{\underset{\text{0}}{\sigma }}}\right)}^{-\alpha I_{\text{c}}} \end{array}$$ (18)

Substituting Eqs (17) and (18) into Eq (7) yields:


$$\begin{array}{c} \frac{\partial \varepsilon_{\text{v}}}{\partial t}=-\frac{\text{1}}{\text{1}+e}\frac{\alpha I_{\text{c}}}{\left(\overset{\prime }{\underset{\text{0}}{\sigma }}-\overset{-}{u_{\text{r}}}\right)\text{ln}\text{1}\text{0}}\left(\frac{\partial \overset{-}{u_{\text{r}}}}{\partial t}-\frac{\partial Q}{\partial t}\right) \end{array}$$ (19)

Substituting Eq (19) into Eq (12) gives:


$$\begin{array}{c} \frac{k_{\text{h}}}{a^{\text{2}}\gamma_{\text{w}}F}\frac{\text{1}+e}{\text{1}+e_{\text{0}}}\left(\overset{-}{u_{\text{r}}}-u_{\text{w}}\right)+\frac{\text{1}}{\text{1}+e_{\text{0}}}\frac{\alpha I_{\text{c}}}{\left(\overset{\prime }{\underset{\text{0}}{\sigma }}-\overset{-}{u_{\text{r}}}\right)\text{ln}\text{1}\text{0}}\left(\frac{\partial \overset{-}{u_{\text{r}}}}{\partial t}-\frac{\partial Q}{\partial t}\right)=\frac{\partial }{\partial z}\left(\frac{\text{1}+e_{\text{0}}}{\text{1}+e}\frac{k_{\text{v}}}{\gamma_{\text{w}}}\frac{\partial \overset{-}{u_{\text{r}}}}{\partial z}\right) \end{array}$$ (20)

Let $A_{\text{1}}=\frac{\text{1}}{\text{1}+e_{\text{0}}}\frac{\alpha I_{\text{c}}}{\left(\overset{\prime }{\underset{\text{0}}{\sigma }}-\overset{-}{u_{\text{r}}}\right)\text{ln}\text{1}\text{0}}$;$A_{\text{2}}=\frac{k_{\text{h}}}{a^{\text{2}}\gamma_{\text{w}}F}\frac{\text{1}+e}{\text{1}+e_{\text{0}}}$, then Eq (20) can be rewritten as:


$$\begin{array}{c} A_{\text{2}}\left(\overset{-}{u_{\text{r}}}-u_{\text{w}}\right)+A_{\text{1}}\left(\frac{\partial \overset{-}{u_{\text{r}}}}{\partial t}-\frac{\partial Q}{\partial t}\right)=\frac{\partial }{\partial z}\left(\frac{\text{1}+e_{\text{0}}}{\text{1}+e}\frac{k_{\text{v}}}{\gamma_{\text{w}}}\frac{\partial \overset{-}{u_{\text{r}}}}{\partial z}\right) \end{array}$$ (21)

Let $A_{\text{3}}=\frac{\pi r_{\text{w}}A_{\text{e}}k_{\text{h}}}{F}\frac{\text{1}+e}{\text{1}+e_{\text{0}}}$, then Eq (16) becomes:


$$\begin{array}{c} \frac{\partial }{\partial z}\left(Q_{\text{w}}\frac{\text{1}+e_{\text{0}}}{\text{1}+e}\frac{\partial u_{\text{w}}}{\partial z}\right)+A_{\text{3}}\left(\overset{-}{u_{\text{r}}}-u_{\text{w}}\right)=\text{0} \end{array}$$ (22)

Under the boundary condition *u*_w_ = −*p* | *r* = *r*_w_ at the drainage boundary, we obtain:


$$\begin{array}{c} u_{\text{w}}=-p_{\text{0}}\left(\text{1}-e^{-\kappa t}\right)\left[\text{1}-\left(\text{1}-k_{\text{1}}\right)\frac{z}{L}\right]\cdot \text{exp}\left[k_{\text{2}}\left(r-r_{\text{w}}\right)\right] \end{array}$$ (23)

According to the principle of superposition, the differential equation governing the upper soil layer can be expressed as:


$$\begin{array}{c} \frac{\partial u_{\text{1}}}{\partial r}=\frac{a^{\text{2}}\gamma_{\text{w}}}{\text{2}k_{\text{s}}}\left(\frac{r_{\text{e}}\sqrt{\overset{\text{2}}{\underset{\text{e}}{r}}-\text{1}}}{\sqrt{r^{\text{2}}-\text{1}}}-r\right){\left[\frac{\text{1}+e_{\text{0}}}{\text{1}+e}\frac{\partial }{\partial a}\left(\frac{\text{1}+e_{\text{0}}}{\text{1}+e}\frac{k_{\text{v}}}{\gamma_{\text{w}}}\frac{\partial \overset{-}{u_{\text{r}}}}{\partial a}\right)+\frac{\partial \varepsilon_{\text{v}}}{\partial t}\right]}_{\left(r_{\text{w}}\leq r\leq r_{\text{s}}\right)} \end{array}$$ (24)

$$\begin{array}{c} \frac{\partial u_{\text{1}}}{\partial r}=\frac{a^{\text{2}}\gamma_{\text{w}}}{\text{2}k_{\text{h}}}\left(\frac{r_{\text{e}}\sqrt{\overset{\text{2}}{\underset{\text{e}}{r}}-\text{1}}}{\sqrt{r^{\text{2}}-\text{1}}}-r\right){\left[\frac{\text{1}+e_{\text{0}}}{\text{1}+e}\frac{\partial }{\partial a}\left(\frac{\text{1}+e_{\text{0}}}{\text{1}+e}\frac{k_{\text{v}}}{\gamma_{\text{w}}}\frac{\partial \overset{-}{u_{\text{r}}}}{\partial a}\right)+\frac{\partial \varepsilon_{\text{v}}}{\partial t}\right]}_{\left(r_{\text{w}}\leq r\leq r_{\text{s}}\right)} \end{array}$$ (25)

Where *u*_1_ is the excess pore water pressure in the upper soil layer.

From the derivation, the expression for the average excess pore water pressure *ū*_1_ in the upper soil layer can be obtained as, as shown in Eq (26):


$$\begin{array}{c} \overset{-}{u_{\text{1}}}=-\frac{F}{\pi r_{\text{w}}A_{\text{e}}k_{\text{h}}}\frac{\partial }{\partial \xi }\left(Q_{\text{w}}\frac{\partial u_{\text{w}}}{\partial \xi }\right)-p_{\text{0}}\left(\text{1}-e^{-\kappa t}\right)\left[\text{1}-\left(\text{1}-k_{\text{1}}\right)\frac{z}{L}\right]\cdot \text{exp}\left[k_{\text{2}}\left(r-r_{\text{w}}\right)\right] \end{array}$$ (26)

The expression for the average degree of consolidation in the upper soil layer [30] is:


$$\begin{array}{c} \overset{-}{U_{\text{1}}}=\text{1}-\frac{\text{1}}{L}\int_{\text{0}}^{L}\frac{\overset{-}{u_{\text{1}}}}{p_{\text{0}}}\text{ d}z \end{array}$$ (27)

By combining Eqs (26) and (27), the average degree of consolidation *Ū*_1_ for the upper soil layer can be determined.

For the lower soil layer, the governing equation is:


$$\begin{array}{c} \frac{\partial \overset{-}{u_{\text{2}}}}{\partial t}=C_{\text{v}}\frac{\partial^{\text{2}}\overset{-}{u_{\text{2}}}}{\partial {\text{z}}^{\text{2}}} \end{array}$$ (28)

Where *ū*_2_ is the average excess pore water pressure in the lower soil layer; *C*_v_ is the vertical coefficient of consolidation,$C_{\text{v}}=\frac{k_{\text{v}}E_{\text{s}}}{\gamma_{\text{w}}}$; and *t* is the consolidation time.

According to the expression for average pore water pressure proposed by Xie Kanghe et al. [31], the average excess pore water pressure at any depth within the lower soil layer can be expressed as:


$$\begin{array}{c} \overset{-}{u_{\text{2}}}=\frac{\text{2}}{\text{3}\pi r_{\text{e}}l\text{2}}\int_{F}u_{\text{2}}\text{ d}A \end{array}$$ (29)

Where *F* represents the effective influence zone at the bottom of the drainage plate.

According to the principle of continuity at the drainage interface, it follows that:


$$\begin{array}{c} u_{\text{2}}=p_{\text{0}}e^{-mT_{\text{v}}}+p_{\text{0}} \end{array}$$ (30)

In this expression: *T*_v_ represents the vertical consolidation time factor,$T_{\text{v}}=\frac{C_{\text{v}}t}{l^{\text{2}}}$; and *m* is a boundary parameter.

By combining Eqs (28)–(30) with the boundary conditions and the continuity conditions for flow between upper and lower soil layers:$\frac{\partial \overset{―}{u_{\text{2}}}}{\partial z}{|}_{z=l}=\text{0}$, ${\overset{―}{u_{\text{2}}}}_{\left(z,\text{0}\right)}=\text{0}$, $\frac{\partial u_{\text{2}}}{\partial z}=\text{0}$, the following expression can be obtained:


$$\begin{array}{c} \frac{\partial \overset{-}{u_{\text{2}}}}{\partial T_{\text{v}}}={\left(\text{1}+p_{\text{0}}-\overset{-}{u_{\text{2}}}\right)}^{\text{1}-C_{\text{v}}}\left[p_{\text{0}}l\left(\text{1}-e^{-mT_{\text{v}}}\right)-\overset{-}{u_{\text{2}}}\right] \end{array}$$ (31)

Following the calculation method proposed by Tian Yi et al. [29], Eq (28) can be transformed into a finite difference form:


$$\begin{array}{c} \frac{\overset{i+\text{1}}{\underset{j}{u}}-\overset{i}{\underset{j}{u}}}{\Delta T}={\left(\text{1}+p_{\text{0}}-\overset{i}{\underset{j}{u}}\right)}^{\text{1}-C_{\text{v}}}\left(\overset{i}{\underset{j}{Y}}-\overset{i}{\underset{j}{u}}\right) \end{array}$$ (32)

Where $\overset{i}{\underset{j}{Y}}=p_{\text{0}}l(j-\text{1})\left(\text{1}-e^{-m(i-\text{1})△T}\right)$.

The initial conditions can be expressed as:


$$\begin{array}{c} \overset{\text{1}}{\underset{j}{u}}=p_{\text{0}} \end{array}$$ (33)

According to the definition, the average degree of *t*_i_ consolidation of the lower soil layer at time *Ū*_2_ can be expressed as:


$$\begin{array}{c} \overset{-}{U_{\text{2}}}=\frac{\sum_{i=\text{1}}^{N+\text{1}}\left(p_{\text{0}}-\overset{i}{\underset{j}{u}}\right)}{\sum_{j=\text{1}}^{N+\text{1}}\left(p_{\text{0}}-\overset{\infty }{\underset{j}{Y}}\right)} \end{array}$$ (34)

By combining Eqs (27) and (34), the total average degree of consolidation of the entire foundation *Ū* can be obtained:


$$\begin{array}{c} \overset{-}{U}=\frac{\overset{-}{U_{\text{1}}}L+\overset{-}{U_{\text{2}}}l}{L+l} \end{array}$$ (35)

It should be noted that Eq (27) represents the numerical solution for the consolidation degree of the upper foundation soil, while Eq (34) is the derived expression for the average excess pore water pressure in the lower soil layer. Using the initial condition provided in Eq (33), the average excess pore water pressure at any time and depth within the lower soil layer can be calculated. Subsequently, Eq (35) can be applied to compute the average degree of consolidation for the entire foundation soil.

## Comparative analysis of results

### Comparison with other solutions

**Fig 6:**
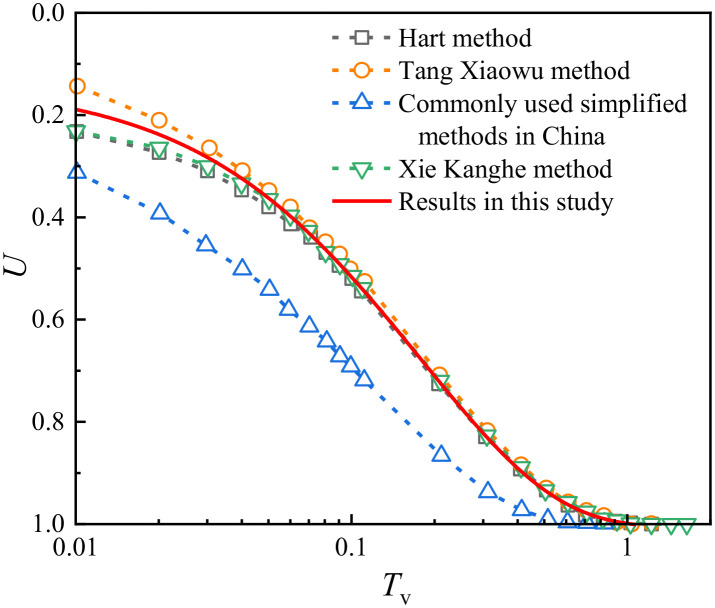
Comparison curves of this paper’s solution with other solutions.

Based on the theoretical derivations presented above, a numerical solution for the large-deformation consolidation model that accounts for the effective influence range at the drainage plate bottom was obtained. To evaluate the accuracy of the proposed model and the validity of the superposition method, comparisons were made with the Hart method [32], commonly used simplified methods in China [33], the Xie Kanghe method [34], and the Tang Xiaowu method [35]. The results are presented in Fig 6. The calculation parameters were adopted from reference [36] as follows:

Fig 6 indicates that commonly used simplified methods in China tend to overestimate the degree of soil consolidation. The Hart method and the Xie Kanghe method yield highly consistent curves throughout the consolidation process, with their results being essentially identical. In contrast, the Tang Xiaowu method exhibits some deviation during the early stage of consolidation, with a difference of approximately 8%, although its predictions converge with the other two methods in the mid-to-late consolidation stages. The results obtained in this study, while lying between the Tang Xiaowu and Xie Kanghe methods in the early stage, show a maximum error of less than 3% (the maximum absolute difference in the calculated degree of consolidation across all evaluated time steps) and similarly converge with these curves in the mid-to-late stages. From a theoretical perspective, the slight divergence observed during the early stages of consolidation is primarily attributed to the inclusion of the effective influence zone at the bottom of the drainage plate and the continuous permeable boundary conditions. This mechanism leads to the intermediate early-stage consolidation rate observed in the present results. To further substantiate this comparison, the Root Mean Square Error (RMSE) was introduced as an additional evaluation index, yielding a value of less than 0.015 between the proposed model and the reference methods, indicating good overall agreement. This demonstrates the reliability of the superposition method employed in this study for calculating soil consolidation and indicates that the proposed numerical solution provides a robust approach for estimating the average degree of consolidation of the entire foundation while accounting for the effective influence range at the drainage plate bottom.

### Comparison with experimental results

**Fig 7:**
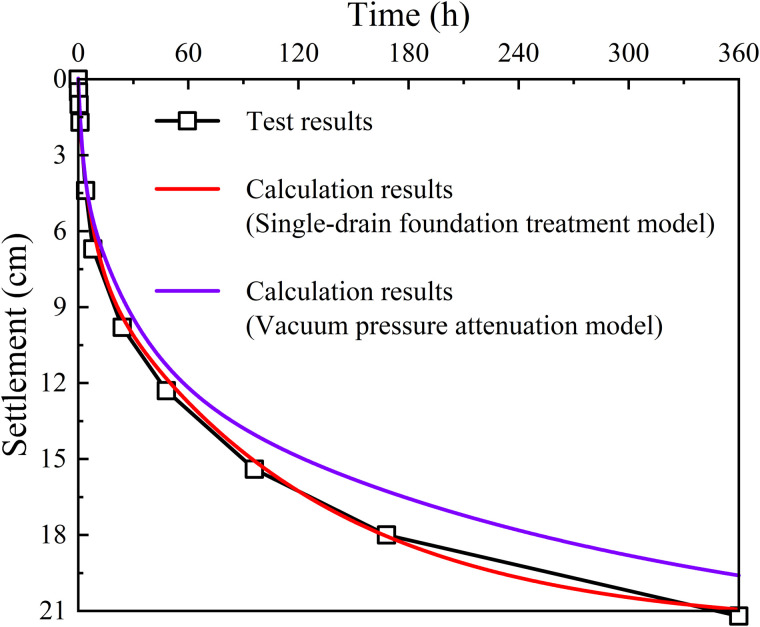
Comparison of the test results with the solution of this paper.

Using experimental data and considering both initial and boundary conditions, the numerical solution proposed in this study was implemented via programming software to determine the temporal variation of surface settlement across the foundation, as shown in Fig 7. The numerical solution exhibits excellent agreement with the experimental results, with a maximum error of less than 3%, further validating the accuracy of the proposed approach. In contrast, the numerical solution of the large-deformation consolidation model that incorporates vacuum loss consistently overestimates settlement relative to the experimental data. This indicates that neglecting the effective influence range at the drainage plate bottom in models accounting for vacuum loss reduces predictive accuracy and underestimates the final settlement of ultra-soft soil foundations, potentially impacting engineering design. Therefore, when evaluating settlement in large-area ultra-soft soil foundations treated with plastic drainage plates combined with vacuum preloading, considering the effective influence range at the drainage plate bottom is both reasonable and necessary. The numerical solution presented in this study provides a practical and reliable tool for predicting settlement in vacuum-preloaded reinforced ultra-soft soil foundations.

Furthermore, it is important to emphasize that the significance of the bottom influence zone in predicting consolidation is highly dependent on the PVD installation depth. In engineering applications where PVDs are installed to relatively shallow depths, the localized consolidation and stress redistribution occurring beneath the drain tip represent a substantial portion of the total volumetric strain. As a result, the proposed model provides a more accurate representation of the early-stage behavior, particularly the rapid initial settlement observed upon vacuum application, which traditional models often underestimate. For significantly deeper installations, while the bottom influence zone still exists physically, its relative contribution to the total macroscopic settlement is proportionally reduced. Therefore, explicitly accounting for the effective influence range at the drainage plate bottom is most critical for ensuring the accuracy of settlement predictions in shallow to moderately deep vacuum preloading treatments of ultra-soft soil foundations.

## Conclusions

Based on the assumptions of the elliptical cylinder equivalent model, this study accounts for nonlinear soil compressibility and permeability characteristics, as well as vacuum pressure that decreases linearly along the PVD depth while varying nonlinearly in the radial soil direction. Using superposition and finite difference methods, a numerical solution for large-deformation consolidation that incorporates the effective influence range at the drainage plate bottom was derived and validated.

1. The model that accounts for the effective influence range at the drainage plate bottom produces results that, while lying between the Tang Xiaowu and Xie Kanghe methods during early consolidation, differ by less than 3% and converge with these methods in the mid-to-late consolidation stages.
2. The calculated curves from this model show excellent agreement with experimental results, with a maximum error below 3%, confirming the validity of both the consolidation model and the proposed numerical solution. In contrast, the numerical solution of the model that considers vacuum loss exhibits larger deviations relative to the model incorporating the bottom influence range.
3. Neglecting the effective influence range at the drainage plate bottom results in smaller predicted foundation settlements, thereby underestimating the final settlement of ultra-soft soil foundations, which may pose risks to the long-term stability and safety of the foundation.

## Supporting information

S1 Dataset (XLSX)

S2 Dataset (XLSX)
